# Emotions towards potential genetic offspring among oocyte donors: a cross-sectional study

**DOI:** 10.1186/s12978-021-01311-8

**Published:** 2021-12-20

**Authors:** Sahar Khosravi, Ashraf Kazemi, Seyyed Mehdi Ahmadi

**Affiliations:** 1 Reproductive Health Department, Isfahan University of Medical Sciences, Isfahan, Iran; 2 Reproductive Health Department, School of Nursing and Midwifery, Isfahan University of Medical Sciences, Hezarjerib AV, Isfahan, Iran; 3 Isfahan Fertility and Infertility Center, Isfahan, Iran

**Keywords:** Oocyte donation, Emotion, Offspring, Assisted reproductive techniques

## Abstract

**Background:**

The presence of maternal emotions towards the offspring resulting from assisted reproductive techniques (ART) has been previously reported in oocyte donors. However, there is limited information about the presence of these emotions in oocyte donors during the ART process and before pregnancy. The aim of this study was to evaluate the emotions of oocyte donor women towards the potential genetic offspring and to compare them with women treated with ART by using own oocytes.

**Methods:**

A cross-sectional study was conducted on 100 women who were divided into two groups of oocyte donors and those treated with ART and using autologous oocyte. At the time of oocyte retrieval. Using a validated questionnaire, the emotions toward potential offspring (EPO) resulting from ART and its three dimensions (including imagination, sense of ownership, and importance of treatment outcome) were measured and compared in two groups.

**Results:**

Comparison of the EPO in the two groups showed that the emotions in all three dimensions were lower in oocyte donors than the other group (p < 0.001). Moreover, in oocyte donors, the mean score of the scale of the importance of treatment outcome dimension was higher than the other two scales (p < 0.001).

**Conclusion:**

The results of the study showed that there is a significant emotion toward the potential offspring in oocyte donors. The presence of these emotions thus should be considered in formulating the ethical charter of ART by using oocyte donation.

## Plain English Summary

There is limited information about the presence of maternal emotions in egg donor women during the assisted reproductive techniques (ART) process and before pregnancy. The aim of this study was to evaluate these emotions of women towards the potential genetic offspring and to compare them with women treated with ART by using own oocytes. A cross-sectional study was conducted on 100 women who were divided into two groups of egg donor and those treated with ART and using autologous oocyte. At the time of oocyte retrieval and using a validated questionnaire, the emotions toward potential offspring (EPO) resulting from ART and its three dimensions (including imagination, sense of ownership, and importance of treatment outcome) were measured and compared in two groups. Out of 100 women. Comparison of the EPO in the two groups showed that the emotions in all three dimensions were lower in egg donors than the other group. Moreover, in egg donors, the mean score of the scale of the importance of treatment outcome dimension was higher than the other two scales. The results of the study showed that there is a significant emotion toward the potential offspring in oocyte donors. The presence of these emotions thus should be considered in formulating the ethical charter of ART by using oocyte donation.

## Background

The widespread use of the third party in assisted reproductive techniques (ART) and the success of infertility treatment following its use has led to an increased number of women volunteers who enter the oocyte donation process [1], mainly for financial reasons and altruism [2, 3]. This process leads to the creation of a creature that is the genetic offspring of the donor and may inherit the thoughts and feelings of the genetic mother.

Willingness to connect with the born offspring in some gamete donors [4], oocyte donors' concerns about the recipient's physical and mental health to ensure the proper development and upbringing of the offspring [5], their concerns about the recipient's social and economic status [6], and the possibility of an emotional bond between the donors and the offspring resulting from this process, and interest in the genetic offspring [7] are among the evidence confirming the existence of these emotions in donors. Moreover, examining the feelings of women who have used a surrogacy and shared their oocytes for the creation of an embryo, studies have shown that these women consider the resulting offspring as their own child [8]. However, unlike the intended mother in surrogacy, oocyte donors are not considered as the mother of the resulting offspring. However, there is evidence showing the maternal emotional feelings of the donors towards the potential genetic offspring resulting from the donation process [3].

It is believed that genetic relationships form the basis of the parent–child relationship [9]. In this regard, research on the views of oocyte donors has shown that donor women considered genetic relationship as an integral part of the family concept and stated that a woman, even if she donates her oocyte to another woman, still considers herself the mother of that child [6]. A qualitative study in Iran showed that oocyte donor considered the offspring as part of their own existence [3].

Although evaluation of oocyte donors' experiences in qualitative studies has shown the presence of emotional responses in donors, the presence of these emotions in oocyte donors towards the potential offspring has not been reported during the ART process and before the confirmation of the clinical pregnancy. However, the recognition of these emotions at the time of the donor's participation in ART allows the development of ethical charter for using a third party in ART. Therefore, the aim of this study was to evaluate the emotions of oocyte donor women towards the potential genetic offspring and to compare them with women treated with ART by using own oocytes.

## Methods

To evaluate the emotions of oocyte donors to the potential offspring this cross-sectional study was performed on 50 oocyte donors and 50 women under ART through using own oocyte with the approval of the ethics committee of Isfahan University in 2019–2020 Isfahan, Iran. A necessary sample size was 50 women in each group in order to achieve a 95% confidence interval and 80% test power to identify significant differences in means values at the 5% level and a significance level alpha of 0.05.

Inclusion criteria in the two groups consisted of having no history of major psychological disorders, sexually transmitted diseases and hepatitis B and C based on the documents recorded in their medical file. For oocyte donor women previous complication with ovarian stimulation and history of genetic diseases were other inclusion criterion. ART cycle cancellation was exclusion criteria for both groups.

Using convenience sampling method, sampling was performed in Isfahan Fertility and Infertility Center before ovarian stimulation among the oocyte donor women and the women who were candidate for ART due to male factor infertility. After obtaining informed consent, the baseline characteristics were recorded and, then, the EPO was measured on the day of oocyte reception and after the operation by using the self-report 12-item emotions towards the potential offspring questionnaire (Table 1). If the participant preferred, the questionnaire was completed through interview. The 12-item questionnaire of emotional response with 5 reversed items had been designed in three dimensions including, the imagination (4 items), sense of ownership (4 items), and importance of treatment outcome (4 items). The items of the questionnaire were designed based on a 4-point Likert scale (0–3) including strongly disagree (0) disagree (1) agree (2) strongly agree (3).

**Table 1:** Emotions towards the potential offspring questionnaire

Domains	Items
Imagination	I think about how much the fetus looks like me
	Only seeing the born baby can excite me (R)
	I can imagine the face of the created fetus
	Seeing the resulted child will excite me
Sense of ownership	I consider the created fetus as part of my existence
	I do not care about the child I have never seen (R)
	That a fetus is the result of my oocyte does not create a special feeling in me (R)
	To be interested in a fetus, I need to grow it in my womb (R)
Importance of treatment outcome	The future life of the created fetus is important to me
	I will be upset if there is a problem with the fetus during the pregnancy
	After finishing the treatment process, I do not care about the outcome of the pregnancy (R)
	The gender of the fetus resulting from the treatment is important to me

*R* reversed

The score of each domain was the sum of the related item scores of the questionnaire. A higher score in each dimension and total scale reflect more emotion toward potential offspring [10]. The research data and the intended variables were compared in two groups using SPSS software version 19 and statistical methods of t-test, Mann–Whitney, and Chi-square tests. Moreover, the mean scores of different dimensions in each group were compared using paired t-test. The relationship between emotional response and contextual variables was assessed using multivariate linear regression.

## Results

Of the 52 eligible oocyte donor women and 52 women treated with ART by using own oocytes, 50 individuals in oocyte donor group and 51 individuals in own oocyte group accepted to participate in the study. In own oocyte group one woman was excluded from the study that was due to cancelation of the ART cycle. The analysis was performed on data from 50 participants in each group.

Based on the results of the study, the two groups were different in terms of age, education level and employment. The mean age of the oocyte donors was lower than the own oocyte group (28.48 vs. 31.80), while they had lower education level and most of them were employed (Table 2). The results of comparing the mean score of total EPO and its dimensions in the two groups are shown in Table 3. According to the results, both groups had significant maternal emotions, thought the own oocyte group scored higher.

**Table 2:** Comparison of the individual characterizes between two groups

Groups	Mean (SD) or number (%)		Sig
	Oocyte donor	Own oocyte	
Age	28.48 (3.15)	31.80 (4.63)	< 0.001
Education			0.03
Less than high school (%)	13 (23.00)	7 (14.00)	
High school & diploma (%)	24 (49.00)	17 (34.00)	
University degree (%)	14 (28.00)	26 (52.00)	
Occupation status (%)			< 0.001
Employed	27 (54.00)	23 (46.00)	
Monthly income ($)	693.10 (240.21)	1390.21 (121.66)	< 0.001
Number of children			< 0.0001
Zero	11 (22.40)	38 (76.00)	
One	28 (14.00)	7 (57.10)	
Two	9 (18.40)	3 (6.00)	
Three or more	3 (6.00)	0 (0.00)	

There were no missing data

*Sig* significant, *SD* standard deviation

**Table 3:** Comparison of the emotions towards offspring in the two groups

Groups	Mean (SD)		Sig
	Oocyte donor	Own oocyte	
Total emotions towards offspring	39.84 (10.81)	49.52 (3.97)	< 0.001
Imagination	11.76 (5.32)	15.48 (3.12)	< 0.001
Sense of ownership	10.50 (4.79)	14.28 (1.68)	< 0.001
Importance of treatment outcome	17.66 (3.23)	19.86 (0.53)	< 0.001

There were no missing data

*Sig* significant, *SD* standard deviation

Comparison of the mean scores of the three dimensions of EPO shown in the donor women showed that the mean score of importance of treatment outcome dimension was higher than the mean score of imagination dimension (p < 0.001). Also, the mean score of importance of treatment outcome was higher than the mean score of the sense of ownership dimensions in the donor women (p < 0.001).

Also, in the own oocyte women the mean score of importance of treatment outcome dimension was higher than the imagination (p < 0.001) and sense of ownership dimensions (p < 0.001). The mean scores of imagination and sense of ownership did not differ in the oocyte donor women; but in women treated with own oocytes, the mean score of imagination was significantly higher than that of sense of ownership (p = 0.02).

In oocyte donor women, independent of the age, monthly income and educational level, the sense of ownership score was related to the number of children inversely and decreased with the increase of number of children (p-0.005). Additionally, the scores of sense of ownership (p = 0.04) and the importance of treatment outcome (p < 0.001) had a significant inverse relationship with the economic level of the oocyte donor women (Table 4).

**Table 4:** Relations between emotions towards offspring dimensions and demographic profile in the oocyte donors

	Emotions towards offspring											
	Imagination				Sense of ownership				Importance of treatment outcome			
	Adj r^2^ = 0.05; F = 0.39; p = 0.81				Adj r^2^ = 0.25 F = 5.14; p = 0.002				Adj r^2^ = 0.27; F = 4.14; p = 0.006			
	Beta	Sig	CI 95%		Beta	Sig	CI 95%		Beta	Sig	CI 95%	
Age of women	− 0.04	ns	− 0.53	0.41	0.12	ns	− 0.19	0.57	− 0.01	ns	0.97	− 0.28
Monthly income ($)	0.01	ns	− 0.01	0.01	− 0.47	0.04	− 0.01	− 0.01	− 0.49	< 0.001	− 0.01	− 0.04
Education	0.02	ns	− 0.50	0.56	− 0.13	ns	− 0.65	0.21	0.05	ns	0.67	− 0.23
Number of children	− 0.17	ns	− 3.30	0.87	− 0.37	0.005	− 4.22	− 0.79	− 0.20	ns	0.04	− 2.44
Occupation status	− 0.07	ns	− 2.76	1.24	− 0.08	ns	− 2.32	1.09	− 0.09	ns	− 1.69	0.59

*Adj r*^2^ adjusted r square; *CI* confidence interval; *Beta* standardized coefficients

## Discussion

The aim of this study was to evaluate the emotions of oocyte donor women towards the potential genetic offspring and to compare them with women treated with ART by using own oocytes. The results showed that oocyte donor women had a relatively significant emotion towards the potential genetic offspring. Furthermore, while these women were younger and less literate than those treated with ART through using own oocytes, their emotions toward potential offspring response were lower than those of the women undergoing ART with own oocytes.

The results showed that the individual characteristics of the participants were different in the two groups. In ART through using donated oocytes, oocyte donors are selected from among young women in order to obtain an appropriate number of oocytes with high fertility potential and increase the treatment success. Moreover, as studies have shown that the oocyte donor’s participation in ART is mainly because of financial motivations [1, 3, 11], the lower economic condition of this group can be justified.

The finding suggests that prior to embryo formation; the oocyte donor women were somewhat emotionally involved with the potential genetic offspring, reflecting maternal attitudes toward the offspring resulting from the ART. The emotions of the oocyte donor women after the end of the participation in ART and the occurrence of pregnancy have been reported in previous studies. According to these studies, before starting the oocyte donation process, some women have expressed their concerns about the possibility of an emotional bond with the resulting offspring and their fondness for it [7]. Some concerns have also been reported in donors about the future of the resulting offspring. Thus, it has been shown that the need to ensure the proper growth and education of their genetic offspring and the mental and physical health of the oocyte recipients have been the main concerns of the oocyte donors [5]. According to the study conducted by Shaw et al. the oocyte donor women believe that the concept of genetic connection means the right to have contact with the resulting offspring [12]. It was shown in this study that donors considered the genetic connection between parents and children to be effective in shaping the family structure and believed that this connection is an integral part of the concept of family. According to this report, even after the donation process, the donors identified themselves as the mother of the resulting offspring and had a feeling of ownership to them [6]. These reports suggest the presence of the maternal emotions of the oocyte donors towards the resulting offspring. The present study, not only confirmed these results, but also showed that maternal emotions exist in these women at the time of participation in ART even before the formation of the fetus. In Stainton's study, parents imagined their fetus before fertilization and even when they were planning for pregnancy [13].

Imagination of the offspring is one of the events that take place during the bonding of the mother with the fetus [14]. Additionally, genetic linkage has been shown to lead to the formation of claims of belonging [15]. These results suggest that genetic mothers considered an identity for their potential embryos resulting from their donated oocyte and imagined it in their mind. However, another finding of the study showed that these emotions in the oocyte donors are not as strong as in women undergoing ART using own oocytes. This research finding indicates that the donors' lack of legal ownership of their offspring does not completely prevent them from expressing their maternal emotions.

The higher level of the dimension of the importance of treatment outcome compared to the other two dimensions of imagination, sense of ownership of the potential offspring, shows that the condition in which the child will grow up and the future that awaits him are so important for the oocyte donors. This research finding is in line with a study by Purewal et al. suggesting that it is important for the oocyte donors to have access to the information such as the number of the children, the history of divorce and socio-economic status of recipient couples [16].

Another finding of the study showed that women with lower economic status and more children experience fewer emotions when participating in ART. Other studies have shown that financial incentives for oocyte donation are significantly related to the low economic status [17]. Therefore, non-financial motivations may be an important factor in the development of maternal emotions in oocyte donors. Moreover, the relationship between the donors' number of children and their emotions to the potential offspring confirms the need to limit the participation of childless women or those with only one child in ART through oocyte donation. Therefore, it is necessary to consider the expectations of oocyte donors about the parents of the resulting offspring in the initial consultation with them.

Although the results of the present study indicated the presence of maternal emotions of oocyte donors towards potential genetic offspring, its limitations should be considered in interpreting the results of the present study. The first limitation of the study was related to the time of assessing the emotions of the donors. Owing to the researcher's lack of access to the donors, this assessment was performed after their participation in the treatment process on the day of ovulation and before sperm insemination and embryo formation.

Also, in this study the results have been adjusted for some potential confounding factors which may influence the overall score of EPO, however there could still be residual variables which are not considered. Therefore, in-depth understanding on the emotional aspects can be investigated further through qualitative approach. Not following-up of the donors and evaluating their changes over time was another limitation of the present research. Furthermore, since the sampling method wasn’t a random sampling, the sample to generalize to the population might not accurately represent the population.

Another limitation of the study was using a forced Likert scale and limitations of this method to deal the neutral value among respondents who don't have clear response to the item. Despite the limitations of the present study, its results can be helpful in development of the ethical charter in providing ART services through using donated oocytes. It is also suggested that the motivation and emotions of the oocyte donor towards the resulting offspring be considered before they enter the treatment process. Moreover, for a deeper understanding of these emotions in oocyte donors, it is suggested that future researchers compare the emotions of oocyte donors with that of the oocyte recipients.

## Conclusion

The results of the present study showed that albeit the EPO score in oocyte donor women was lower than women treated with own oocyte, the donor group score was high, This results revealed that the oocyte donors were involved in significant emotions towards the potential genetic offspring who does not belong to them and should be taken into account to develop the ethical charter in providing ART services through using donated oocytes.

## Data Availability

Data and material are available on request from the corresponding author.

## Abbreviations

ART: Assisted reproductive techniques
EPO: Emotions toward potential offspring
EPOQ: Emotions toward potential offspring questionnaire
